# A Comprehensive Study of Reactive Oxygen Species Explicit Dosimetry for Pleural Photodynamic Therapy

**DOI:** 10.3390/antiox13121436

**Published:** 2024-11-22

**Authors:** Hongjing Sun, Yihong Ong, Michele M. Kim, Andreea Dimofte, Sunil Singhal, Keith A. Cengel, Arjun G. Yodh, Timothy C. Zhu

**Affiliations:** 1 Department of Radiation Oncology, University of Pennsylvania, Philadelphia, PA 19104, USA; hongjing.sun@pennmedicine.upenn.edu (H.S.); ongyihong@gmail.com (Y.O.); michele.kim@pennmedicine.upenn.edu (M.M.K.); andreea.dimofte@pennmedicine.upenn.edu (A.D.); keith.cengel@pennmedicine.upenn.edu (K.A.C.); 2 Department of Bioengineering, University of Pennsylvania, Philadelphia, PA 19104, USA; 3 Department of Surgery, University of Pennsylvania, Philadelphia, PA 19104, USA; sunil.singhal@pennmedicine.upenn.edu; 4 Department of Physics and Astronomy, University of Pennsylvania, Philadelphia, PA 19104, USA; yodh@physics.upenn.edu

**Keywords:** Reactive oxygen species (ROS), photodynamic therapy dosimetry, diffuse correlation spectroscopy

## Abstract

Photodynamic therapy (PDT) relies on the interactions between light, photosensitizers, and tissue oxygen to produce cytotoxic reactive oxygen species (ROS), primarily singlet oxygen (^1^O_2_) through Type II photochemical reactions, along with superoxide anion radicals (O_2_^•−^), hydrogen peroxide (H_2_O_2_), and hydroxyl radicals (^•^OH) through Type I mechanisms. Accurate dosimetry, accounting for all three components, is crucial for predicting and optimizing PDT outcomes. Conventional dosimetry tracks only light fluence rate and photosensitizer concentration, neglecting the role of tissue oxygenation. Reactive oxygen species explicit dosimetry (ROSED) quantifies the reacted oxygen species concentration ([ROS]_rx_) by explicit measurements of light fluence (rate), photosensitizer concentration, and tissue oxygen concentration. Here we determine tissue oxygenation from non-invasive diffuse correlation spectroscopy (DCS) measurement of tumor blood flow using a conversion factor established preclinically. In this study, we have enrolled 24 pleural PDT patients into the study. Of these patients, we are able to obtain data on 20. Explicit dosimetry of light fluence, Photofrin concentration, and tissue oxygenation concentrations were integrated into the ROSED model to calculate [ROS]_rx_ across multiple sites inside the pleural cavity and among different patients. Large inter- and intra-patient heterogeneities in [ROS]_rx_ were observed, despite identical 60 J/cm^2^ light doses, with mean [ROS]_rx,meas_ of 0.56 ± 0.26 mM for 13 patients with 21 sites, and [ROS]_rx,calc1_ of 0.48 ± 0.23 mM for 20 patients with 76 sites. This study presented the first comprehensive analysis of clinical ROSED in pleural mesothelioma patients, providing valuable data on future ROSED based pleural PDT that can potentially produce uniform ROS and thus improve the PDT efficacy for Photofrin-mediated pleural PDT.

## 1. Introduction

Photodynamic therapy (PDT) is an emerging cancer treatment modality that holds great promise due to its minimally invasive nature, low systemic toxicity, and ability to selectively target tumor cells [1,2,3,4]. PDT involves the administration of a photosensitizing drug that preferentially accumulates in malignant tissues. Upon activation by specific wavelengths of light, the photosensitizer initiates photochemical reactions with molecular oxygen, generating cytotoxic reactive oxygen species (ROS) that destroy tumor cells through oxidative damage mechanisms [5,6,7,8]. PDT can generate various reactive oxygen species through both Type I and Type II photochemical pathways. Our ROSED measurements are primarily composed of singlet oxygen (^1^O_2_) production through Type II mechanisms because Photofrin is a Type II sensitizer. Type II reactions occur when the excited photosensitizer transfers energy directly to ground-state molecular oxygen, producing singlet oxygen. Simultaneously, Type I reactions can generate other ROS including superoxide anion radicals (O2^•−^), hydrogen peroxide (H_2_O_2_), and hydroxyl radicals (^•^OH) through electron transfer processes. The relative contribution of Type I versus Type II mechanisms depends on multiple factors including the photosensitizer type, substrate availability, and local oxygen concentration. For Photofrin-mediated PDT, Type II processes generating singlet oxygen are considered the dominant cytotoxic mechanism, though contributions from Type I ROS cannot be excluded [9,10].

However, realizing the full therapeutic benefit of PDT hinges critically on the delivery of an appropriate light dose in regions containing sufficient concentrations of both the photosensitizing drug and molecular oxygen. If any one of these three essential components is suboptimal, it can severely compromise treatment efficacy. Accurate and comprehensive dosimetry, accounting for the complex interplay among photosensitizer concentration, light fluence, and tissue oxygenation status, is crucial for predicting and optimizing long-term PDT outcomes. Underestimating the concentration of reactive oxygen species ([ROS]_rx_) reacted with the tumor can lead to inadequate cell killing, increasing the risk of tumor recurrence or metastasis. Conversely, overestimating the [ROS]_rx_ exposes normal tissue to unnecessary phototoxic effects, causing collateral damage [11,12,13].

Current state-of-the-art clinical PDT protocols predominantly focus on monitoring PDT dose, i.e., the photosensitizer drug uptake in tissue and the total light fluence delivered during treatment [14]. While straightforward to measure, these metrics alone provide an incomplete picture, as they neglect the critical role of tissue oxygenation status. This is a major oversimplification, as the photochemical mechanisms underlying PDT rely on molecular oxygen as a precursor [15]. Areas with low photosensitizer concentration or oxygen deprivation within the tumor can remain undertreated despite adequate light delivery to those regions on a macroscopic level. Tumor hypoxia, in particular, poses a significant challenge, as it is a common phenomenon induced by the altered metabolic demands and defective vascular biology of malignant tissues. PDT itself can exacerbate hypoxia through consumption of ground-state oxygen and vasculature shutdown effects.

The complex pharmacokinetics influencing heterogeneous photosensitizer distribution, coupled with the spatial and temporal variations in tissue oxygenation induced by vascular PDT effects, severely limit the accuracy of dosimetry metrics based solely on light fluence. Explicit measurements incorporating all three key PDT components are needed for personalized dosimetry and intelligent treatment planning. To address these limitations, strategies for “explicit” dosimetry have been proposed to quantify the reacted singlet oxygen concentration ([ROS]_rx_) as a more biologically-relevant dosimetric quantity [16,17]. By combining voxel-by-voxel mapping of the photosensitizer concentration ([S_0_]), treatment light fluence rate (ϕ) and, crucially, the oxygen level ([^3^O_2_]) within the tumor region, these explicit models enable prediction of the spatial [ROS]_rx_ distribution, which can then guide light dose painting and potentially correlate with local tumor control. Preclinical studies in animal models have demonstrated strong correlations between calculated [ROS]_rx_ and long-term PDT treatment outcomes, positioning explicit ROS dosimetry as a powerful tool for treatment planning and optimization [18]. However, clinical implementation has been hindered by the lack of practical methodologies to non-invasively measure real-time, intra-tumoral oxygen fluctuations during the PDT procedure. This study explores overcoming this key barrier through an innovative approach that derives comprehensive oxygenation information inside the pleural cavity by monitoring tumor hemodynamics and blood flow using diffuse correlation spectroscopy (DCS). When integrated with parallel measurements of light fluence rate and photosensitizer biodistribution, this multimodal platform enables explicit dosimetry of [ROS]_rx_ based on all three governing PDT parameters in an actual patient setting. Implementing comprehensive [ROS]_rx_ mapping has the potential to dramatically improve the level of biologically-relevant individualization and treatment planning achievable for PDT. The resulting dosimetry can guide light dose painting to ensure cytotoxic singlet oxygen levels are reached throughout the entire tumor volume, while sparing surrounding normal tissues. This could significantly enhance the therapeutic ratio and overall efficacy of PDT for a wide variety of solid tumor indications [19,20]. This is the first comprehensive analysis of the effectiveness of modeling vs. measured [ROS]_rx_ in vivo, and we have successfully proposed, for the first time, an improved empirical theory to analyze [ROS]_rx_ based on measured photosensitizer uptake and light fluence alone.

## 2. Materials and Methods

### 2.1. Patient Selection and Treatment Protocol

This study was conducted as part of an ongoing phase II clinical trial evaluating interoperative Photofrin-mediated pleural PDT following surgical tumor debulking for malignant pleural mesothelioma at the University of Pennsylvania. Patients with pathologically confirmed epithelioid mesothelioma were enrolled after providing informed consent under an institutional review board approved protocol (IRB protocol number 819186) (Supplementary Materials).

**Figure 1:**
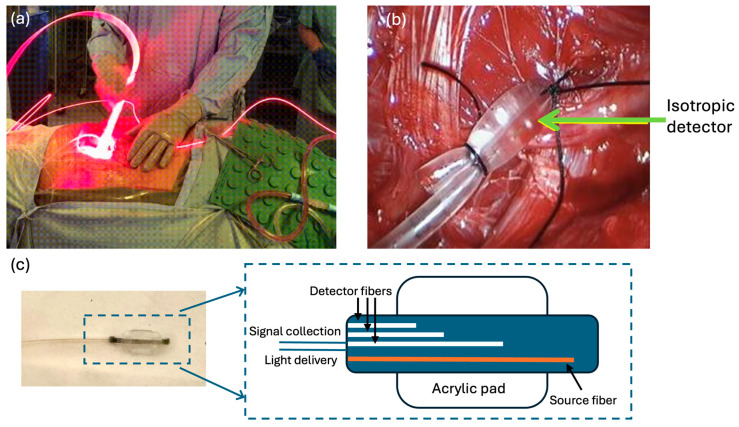
(**a**) Clinical setting during pleural PDT and (**b**) PDT dose (light + PDTuptake) measurement setup, showing the positioning of isotropic detectors and light delivery within the pleural cavity. A 6W fiber optic probe emitting 630 nm laser treatment light is inserted and continuously moved around to uniformly illuminate the inner pleural surface. (**c**) DCS contact probe placement for non-invasive measurement of tissue oxygenation through blood flow monitoring. This innovative approach uses diffuse correlation spectroscopy to derive comprehensive oxygenation information inside the pleural cavity by monitoring tumor hemodynamics and blood flow.

The clinically approved photosensitizer Photofrin (Pinnacle Biologics, Chicago, IL, USA) was administered intravenously at 2 mg/kg approximately 24 h prior to the anticipated PDT treatment time. This drug–light interval allows for adequate accumulation of the photosensitizer in malignant tissues. During surgery, the pleural tumor was resected to the maximal extent possible through an extrapleural pneumonectomy or pleurectomy/decortication procedure. Following gross total tumor debulking, the patient underwent the PDT treatment phase. The lung cavity was filled with a diluted intralipid solution to aid in light diffusion and scattering within the tissue. A fiber optic probe emitting 630 nm laser light (Modulight, Tampere, Finland) was inserted and continuously moved around to uniformly illuminate the inner pleural surface (Figure 1). PDT light was delivered at a prescription of 60 J/cm^2^ surface fluence, as measured by detectors sutured to the chest wall, as described in the next section.

### 2.2. PDT Dose Dosimetry System

An innovative eight-channel dosimetry system featuring isotropic detectors (Medlight SA, Ecublens, Switzerland) was utilized to quantify two of the three key PDT parameters—light fluence rate and photosensitizer concentration—enabling an essential PDT quantity, PDT dose, to be calculated. PDT dose, *D (*μM*·*J*/*cm^2^), is defined by the equation,

(1) $$D=\int_{t=0}^{T}\phi \times \left[S_{0}\right]dt.$$

**Figure 2:**
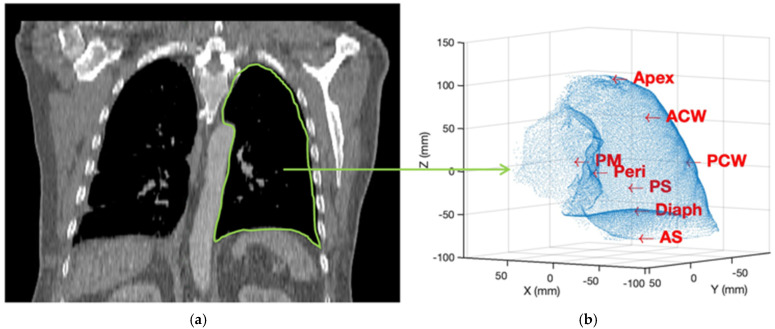
Schematic of detector locations (8) used for each pleural PDT patient: (**a**) Pre-PDT CT scan showing the anatomy of the pleural cavity. (**b**) Illustration of the eight isotropic detector locations on the pleural cavity during PDT. The detectors are strategically placed to cover key areas including the posterior mediastinum (PM), pericardium (Peri), apex, anterior/posterior chest walls (ACW/PCW), anterior/posterior sulci (AS/PS), and diaphragm (Diaph). This multi-point monitoring system provides comprehensive spatial sampling of the PDT dose deposited throughout the complex geometry of the lung cavity during PDT light delivery.

This marked a significant advancement over previous dosimetry technologies limited to single-point monitoring. The dosimetry instrument consisted of eight separate channels, each connected to an isotropic detector that was sutured to a strategic location within the pleural cavity walls (posterior mediastinum, pericardium, apex, anterior/posterior chest walls, anterior/posterior sulci, and diaphragm), as shown in Figure 2. This detector positioning aimed to provide comprehensive spatial sampling of the dose deposited throughout the complex geometry of the lung cavity during PDT light delivery. The isotropic detectors employed in this system are specialized devices designed to measure fluence rate in a nearly 4π collecting solid angle, mitigating errors from detection angle dependencies. Each unit consists of an isotropic spherical probe coupled to optical fibers that carry the transmitted light signals. Within the dosimetry instrument, the optical fibers from each of the eight isotropic detectors were connected to individual channels. These channels split the light signals through internal bifurcated fiber optic cables, allowing simultaneous monitoring of two distinct characteristics.

One branch of the bifurcated fibers from each channel was directed to a photodiode that measured the instantaneous treatment light fluence rate at the corresponding isotropic detector location. The cumulative light fluence delivered to each of the eight sites was calculated in real-time by integrating these fluence rate signals over time. PDT light delivery was terminated upon all eight detectors when the prescription of 60 J/cm^2^ surface fluence was reached. The other branch carried the light to a spectrometer that captured the full fluorescence emission spectrum excited by the PDT treatment light in the surrounding tissue region. These fluorescence signals contained contributions from the administered Photofrin photosensitizer drug, making it possible to quantify the local Photofrin concentration at each of the eight measurement sites. The detailed schematic can be found elsewhere [14]. However, accurately extracting the Photofrin concentration from the raw fluorescence spectra required additional corrections to account for distortions induced by the specific optical properties (absorption and scattering) of the pleural tissue at each location [21,22]. This was achieved through an optical property correction factor derived from diffuse reflectance spectroscopy measurements on the pleural cavity wall before and after PDT [14], calculated as below,

(2) $$CF=25.49\left(\mu_{a}^{0.902}{\mu ’}_{s}^{-1.094}+0.016\right),$$

where *µ_a_* and *µ_s_*’ were the absorption and scattering coefficients.

By networking eight isotropic detector channels in parallel, each with bifurcated monitoring of fluence rate and fluorescence emission spectra, this innovative dosimetry platform provided the first comprehensive spatial mapping of PDT, utilizing two out of the three key PDT parameters—light fluence and photosensitizer concentration. Integrating this with the supplemental diffuse correlation spectroscopy data enabled explicit dosimetry accounting for all three governing components.

### 2.3. Diffuse Correlation Spectroscopy for Oxygen Monitoring

The PDT dose dosimetry system provided measurements of the light fluence rate and photosensitizer concentration, which are two critical parameters required for calculating the ROSED during PDT. However, an additional modality was necessary to quantify the third key parameter: tissue oxygenation levels within the pleural cavity during the treatment. While the direct measurement of oxygen partial pressure (pO_2_) using an oxygen-only bare-fiber sensor (phosphorescence-based, NX-BF/O/E, Oxford Optonix, Oxford, UK) was feasible in preliminary studies, this method was not validated for clinical use [18,23]. This study employed DCS, a non-invasive optical technique that can quantify blood flow in deep tissues by analyzing temporal fluctuations in diffusely scattered light caused by the motion of red blood cells [24,25]. A custom-built DCS probe, consisting of source and detector fibers, was sutured adjacent to one or two of the isotropic detectors at the pleural cavity wall, depending on the clinical conditions. A detailed description of the DCS instrument can be found elsewhere [26].

The DCS system operated in the near-infrared region to avoid interference from the PDT treatment light. It continuously monitored the blood flow index (BFI) at the measurement site throughout PDT delivery. This BFI data was then scaled to approximate [^3^O_2_] using a conversion factor of 1.5 × 10^9^ μMs/cm^2^ [27]. This value was determined through preclinical validation studies directly comparing DCS measurements to invasive oxygen probe readings in mouse tumors undergoing PDT. For further optimization, an oxygen concentration correction factor was applied in the post-treatment processing stage based on the definition *CF_O_*_2_ = [*ROS*]_rx_/*ξ·D*, where *D* is the PDT dose (see Equations (1) and (7)):

(3) CFO2=1∫0TϕtS0dt∫0TO32tϕtS0O32t+βdt

When inserting the [^3^O_2_] and [*S*_0_] with the measured and calculated values, one gets *CF_O_*_2,meas_ = $\frac{{\left[ROS\right]}_{rx,meas}}{\xi \cdot D_{meas}\;}$ and *CF_O_*_2,calc2_ = $\frac{{\left[ROS\right]}_{rx,calc2}}{\xi \cdot D_{calc2}\;}$, respectively, *ϕ* is always a measured value. For calculating *CF_O_*_2,meas_, one can use the fact that the Photofrin concentration, [*S*_0_], does not change over time based on measurement [14], thus *CF*_O_2___,meas_ = 1∫0Tϕtdt·∫0TO23tϕtO23t+βdt. For patients where staffing and logistics permitted, a second DCS probe was utilized to simultaneously monitor oxygenation fluctuations at an additional pleural site. For a subset of 13 patients, distinct DCS measurements were made using this multimodal dosimetry platform. For calculating *CF_O_*_2,calc2_ using Equation (3), our macroscopic model involves a varying [*S*_0_], necessitating its inclusion in the equation. While the current approach uses a standardized initial oxygen concentration of 40 μM, future implementations could benefit from patient-specific oxygen uptake measurements prior to treatment. Such pre-treatment calibration could account for individual variations in baseline tissue oxygenation and potentially improve the accuracy of our oxygen concentration estimates derived from DCS measurements.

### 2.4. Light Fluence Distribution Mapping Integration

While the current dosimetry platform provides comprehensive monitoring of light fluence, photosensitizer concentration, and oxygen levels at discrete measurement sites, a planned future integration aims to incorporate high-resolution light fluence distribution mapping across the entire pleural cavity surface. This will enable unprecedented spatial dosimetry compared to traditional single-point techniques. The proposed approach involves interfacing the eight-channel isotropic detector dosimeter with an innovative infrared (IR) camera (Polaris, NDI, Waterloo, ON, Canada) and 3D surface scanning system developed through parallel research efforts. Prior to the treatment, a handheld 3D scanner (Artec Leo, Senningerberg, Luxembourg) is used to rapidly acquire the complex 3D geometry of the inner pleural cavity surface in a panoramic model. The navigation component utilizes an IR camera to continuously track the real-time 3D position of the PDT light source fiber optic by detecting reflections from passive spherical markers on the treatment wand.

Advanced computational light modeling algorithms will fuse the tracked light source positioning data from the navigation system with the panoramically reconstructed cavity surface model. This permits the calculation of the precise light fluence deposited at every micro-voxel throughout the entire treatment volume on a real-time basis. Fluence modeling accounts for both the direct line-of-sight illumination component from the light source position, as well as the contribution of scattered fluence within the cavity,

(4) $$\phi =\frac{S}{4\pi r^{2}}+b,$$

where *S* is the source power (mW), *r* is the distance (mm) between the point light source and the cavity surface, and *b* is an empirical constant [28]. By generating real-time 2D fluence distribution maps, updated as the light source is moved around, this system will allow identifying over-treated “hot spots” receiving excess light dose as well as under-dosed “cold areas” in need of additional illumination. This voxel-level fluence data can be co-registered and combined with the discrete dosimetry measurements from the isotropic detector locations to quantify heterogeneities, validate calculated values, and continuously monitor and adaptively guide the light painting process to improve uniformity across the entire target region.

The integrated surgical navigation, 3D scanning, light modeling, and multi-channel dosimetry platform represents a groundbreaking technological advance for comprehensive spatial dosimetry and treatment guidance in PDT. Providing interventional physicians with this unprecedented level of real-time feedback on the light fluence distribution during the procedure has the potential to significantly enhance treatment efficacy while reducing collateral toxicities to surrounding normal tissues. Intelligent planning and light painting capabilities could dramatically improve the therapeutic ratio achievable with PDT for a variety of solid tumor indications.

### 2.5. Explicit ROS Dosimetry Calculations

The three explicit dosimetry data streams—ϕ, [*S*_0_], and [^3^O_2_], derived from DCS tumor blood flow monitoring—were integrated into the reactive oxygen species explicit dosimetry (ROSED) model to calculate the reacted singlet oxygen concentration ([ROS]rx) generated during PDT. The ROSED model is based on a set of coupled photochemical kinetic equations that describe the interactions between the photosensitizer ground state, excited states, oxygen, and generation of cytotoxic singlet oxygen. For Photofrin-mediated PDT, the key equations are:

(5) dS0dt=−O23O23+βS0+δϕS0ξσ, 

(6) dO23dt=−O23O23+βϕS0ξ+g1−O23O230, 

(7) d[ROS]rxdt=ξO23O23+βϕS0.

where σ, g, δ, *ξ*, and β are photosensitizer-specific parameters. The values were adopted from previous studies characterizing Photofrin photochemistry, presented in Table 1.

With Equations (1) and (3), the [ROS]_rx_ can also be calculated as the following equation,

(8) $$[ROS{]}_{rx,\;calc1}=\xi \cdot {CF}_{O_{2},mean}\cdot D.$$

(9) $$[ROS{]}_{rx,\;calc2}=\xi \cdot {CF}_{O_{2},calc2}\cdot D.$$

(10) $$[ROS{]}_{rx,meas}=\xi \cdot {CF}_{O_{2},meas}\cdot D.$$

where *D* is the PDT dose (in unit of μM·J/cm^2^). The differential equation (Equation (7)) was numerically integrated over the full treatment time to compute the cumulative [ROS]_rx,meas_ based on the measured time-resolved φ, [*S*_0_], and [^3^O_2_] values at each pleural cavity location monitored by the isotropic detectors and DCS probes. The availability of monitored sites for each clinical case is summarized in Table 2. As illustrated by the table, there were instances where DCS probe measurement was unavailable. To address this need, we developed two distinct methods in this study. Specifically, method 1 was employed to calculate ${CF}_{O_{2},calc1}$ based on the average value of ${CF}_{O_{2},meas}$, ${CF}_{O_{2},mean}$ (see Section 3.2). Method 2 involved simulating ${CF}_{O_{2},calc2}$ using a macroscopic singlet oxygen modeling and Equations (5)–(7), based on the measured ϕ assuming initial oxygen concentration of 40 μM [17]. The calculations effectively mapped the reacted singlet oxygen dose deposited in a patient-specific, intra-operative manner throughout the pleural PDT treatment. The resulting [ROS]_rx_ distribution could then be analyzed for heterogeneities arising from variations in photosensitizer uptake and oxygenation status within the pleural cavity and between different patients receiving the same prescribed 60 J/cm^2^ light dose.

## 3. Results

### 3.1. Photosensitizer Concentration Monitoring

Simultaneously with light fluence and oxygenation monitoring, the comprehensive dosimetry platform tracked the third key PDT parameter—photosensitizer concentration—by capturing fluorescence emission spectra from the Photofrin drug excited by the treatment light. As demonstrated by the previous study, the measured Photofrin levels exhibited remarkable stability at each individual measurement site throughout the entire light delivery period [14]. No significant photobleaching of the photosensitizer was observed. However, in contrast to the temporal stability, substantial heterogeneities in the absolute Photofrin concentrations were evident between different pleural cavity locations, both within the same patient and across patients. The maximum inter-patient variation in administered Photofrin concentrations was 9.2-fold. The maximum intra-patient variation was lower at 3.4-fold. Across all treatment sites, the mean Photofrin concentration was 4.98 μM, and the mean PDT dose delivered was 493.2 μMJ/cm^2^. This agrees with previous evidence that the biodistribution and pharmacokinetics of photosensitizer drugs can be highly heterogeneous, especially in complex tumor environments [29,30].

### 3.2. Tumor Oxygenation Monitoring

**Figure 3:**
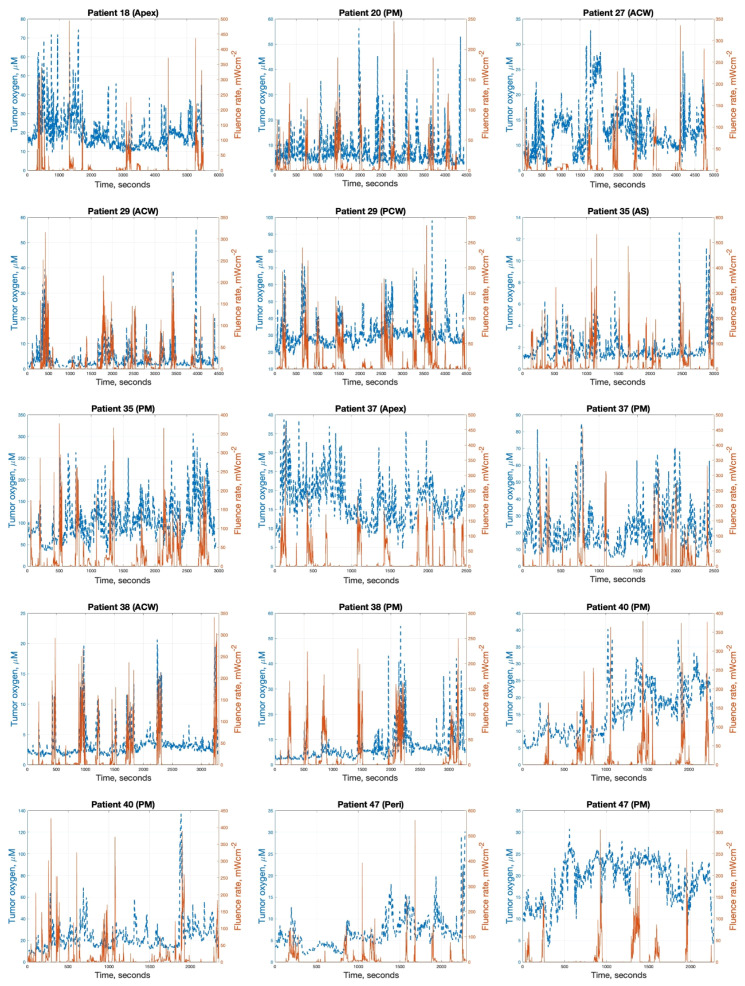

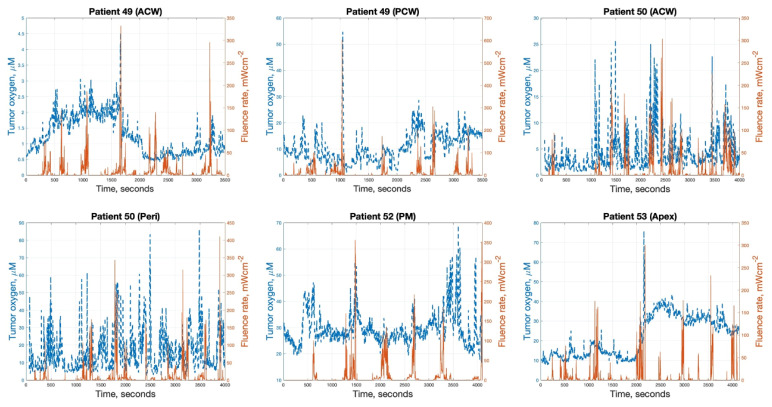
Overlay plots of fluence rate (red solid curve) and oxygen measurement (blue dashed curve) over time for all sites with oxygen concentration measured (13 patients). These graphs demonstrate the dynamic relationship between light delivery and tissue oxygenation during PDT, showing how oxygen levels fluctuate in response to changes in light fluence rate throughout the treatment. Generally, tumor oxygenation is low at the beginning of light delivery due to disruptions in blood flow from the preceding surgical tumor debulking procedure. When high light fluences are applied, an acute reoxygenation response is observed. During periods of low fluence, oxygen concentration decreases back towards initial hypoxic baseline levels.

In addition to PDT dose tracking, the dosimetry platform utilized DCS to monitor fluctuations in tissue oxygenation levels throughout the PDT light delivery process, as depicted in Figure 3 for all sites and patients with available measured oxygen concentrations. They exhibited substantial temporal variations directly correlated with the light fluence rate.

In general, tumor oxygenation was low at the beginning of light delivery due to disruptions in blood flow induced by the preceding surgical tumor debulking procedure. However, as soon as high light fluences were applied, an acute reoxygenation response was observed, with oxygen levels sharply increasing likely due to photochemical mechanisms such as nitric oxide production and microvascular dilation effects. Conversely, during periods of low fluence when the light source was repositioned to other cavity regions, the oxygen concentration would decrease back towards the initial hypoxic baseline levels. This cycling between hyperoxygenation and hypoxia continued throughout the entire light delivery period. The ability to continuously monitor these dynamic oxygenation fluctuations in real time represented a critical advancement compared to traditional PDT dosimetry that assumes constant tumor oxygenation.

**Figure 4:**
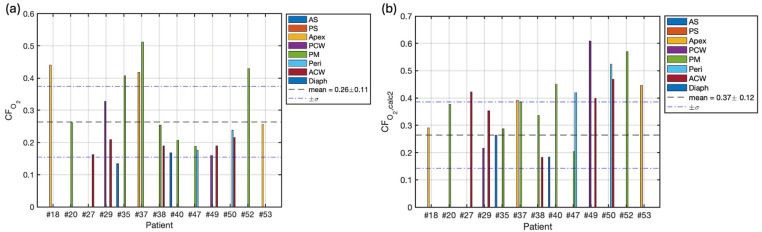
(**a**) Oxygen correction factor ${CF}_{O_{2}}$ per patient for 13 cases using Equation (3) based on oxygen measurements. The graph shows the variability in oxygen correction factors both between patients and across different measurement sites within individual patients. The maximum inter-patient variation was 3.8-fold, while the maximum intra-patient variation was 3.0-fold. The mean ${CF}_{O_{2}}$ across all treatment sites was 0.26 ± 0.11. (**b**) Oxygen correction factor ${CF}_{O_{2},calc2}$ calculated using Equation (3) for comparison, with a mean of 0.37 ± 0.11 across all treatment sites. The mean and standard deviation are represented by black dashed and blue dash-dotted lines, respectively. These correction factors are crucial for accurately calculating the reacted singlet oxygen concentration.

The correction factors for oxygen concentration, ${CF}_{O_{2}}$, for 13 cases calculated by Equation (3) are summarized in Figure 4a. Variations were observed in the treatment response, with a maximum inter-patient variation of 3.8-fold and a maximum intra-patient variation of 3.0-fold in the administered photosensitizer concentrations. The mean ${CF}_{O_{2}}$ across all treatment sites was 0.26 ± 0.11. For comparison, Figure 4b presents the ${CF}_{O_{2}}$ (calc 2) values calculated using our macroscopic model. In this case, the mean across all treatment sites was 0.37 ± 0.11.

### 3.3. Light Fluence Rate Monitoring and COMSOL Multiphysics

**Figure 5:**
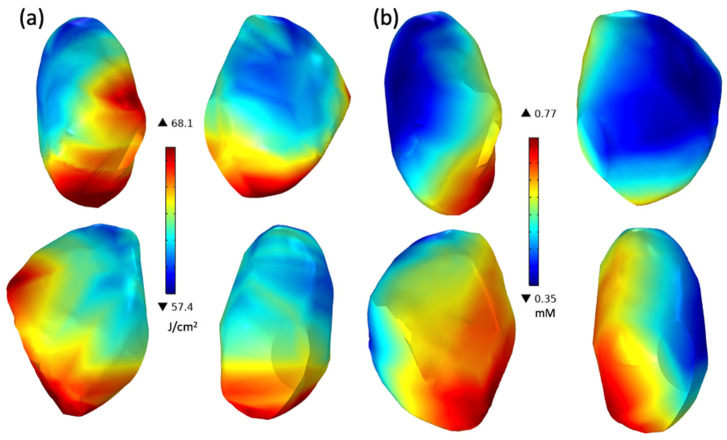
Distribution of (**a**) cumulative light fluence and (**b**) singlet oxygen concentration ([ROS]_rx_) for a representative patient simulated using COMSOL. The resulting [ROS]_rx_ ranges from 0.35 to 0.77 mM, which agrees with the explicit [ROS]_rx_ dosimetry calculation. These images illustrate the heterogeneity in light distribution and reactive oxygen species generation across the pleural cavity surface during PDT.

The clinical PDT dosimetry system enabled real-time tracking of the treatment light fluence rate at eight discrete sites across the pleural cavity during light delivery. This ensured that the prescribed light dose of 60 J/cm^2^ was achieved at all monitored sites by the treatment’s conclusion. Concurrently, an IR camera tracked the point light source’s position throughout the treatment duration. In previous clinical cases, technical difficulties precluded the simultaneous visualization of the measured data, relegating it to post-treatment analysis purposes. The light distribution on the inner cavity surface at the end of treatment, obtained by the IR camera tracking data, for a representative patient is presented in Figure 5a. Despite achieving the described light dose at the locations monitored by the isotropic detector, the overall light distribution exhibited some deficiencies, suggesting regions of both over- and under-dosing. This highlights the potential for further optimization of light delivery to achieve a more uniform dose deposition across the entire treatment volume.

The pleural cavities were reconstructed utilizing the monitored light position data and imported into COMSOL Multiphysics 5.0 for further simulation. Detailed information regarding the tracking system and reconstruction process can be found in previous publications [28]. The delivered fluence values were also imported into COMSOL as 3D fluence clouds and assigned to their respective positions (x, y, and z) on the reconstructed contour. Subsequently, the forward calculation of the macroscopic kinetic Equations (5)–(7) was performed to simulate the reactive oxygen species concentration ([ROS]_rx_) on the surface of the pleural cavity (Figure 5b). For this specific case, the simulated [[ROS]_rx_ ranged from 0.35 mM to 0.77 mM.

### 3.4. Explicit [ROS]_rx_ Dosimetry Calculations

**Figure 6:**
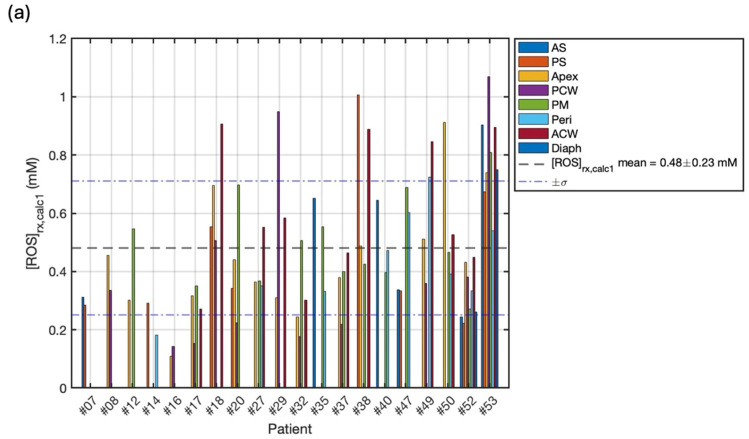

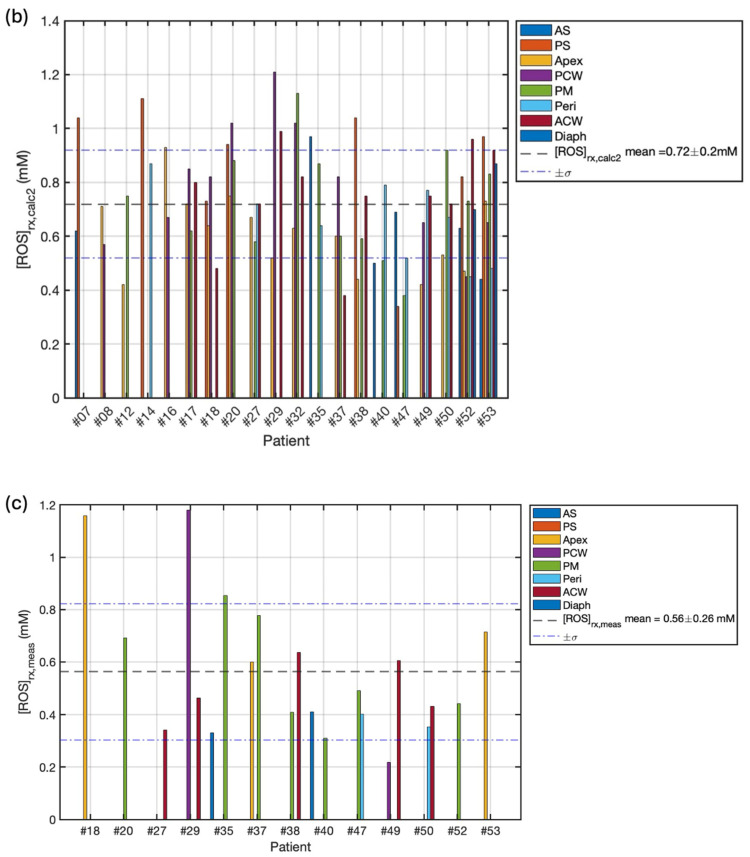
(**a**) Calculated Reactive Oxygen Species concentration, [ROS]_rx,calc1_, per patient for 20 cases calculated by Equation (8), based on average oxygen concentration correction factor, ${CF}_{O_{2}}$ and PDT doeses. (**b**) Reactive Oxygen Species concentration calculated with oxygen concentration simulated using macroscopic model, [ROS]_rx_,_calc2_, per patient for 20 cases calculated by Equation (9), for individual sites and cases. (**c**) Measured Reactive Oxygen Species concentration, [ROS]_rx,meas_, per patient for 13 cases using Equation (10) incorporating measured oxygen measurements. The mean and standard deviation are represented by the black dashed line and blue dash-dotted line, respectively.

By integrating the comprehensive real-time dosimetry data into the ROSED model, the reacted singlet oxygen concentration [ROS]_rx_ could be explicitly calculated as a more biologically-relevant dosimetric quantity directly tied to cytotoxic PDT effects [18]. In cases where direct measurements of oxygen concentration are unavailable, the mean ${CF}_{O_{2},mean}$ and Equation (8) can be utilized to estimate the [ROS]_rx_. To facilitate comparison and validation, this method was employed for all treatment sites, as illustrated in Figure 6a. Across a cohort of 20 patients, the calculated mean [ROS]_rx, calc1_ was found to be 0.48 ± 0.23. To further investigate the possibility of calculating [ROS]_rx_ when direct measurement of [^3^O_2_] is not available, we developed a macroscopic modeling method based on Equations (5)–(7), utilizing light fluence rate measured by isotropic detectors, as shown by the red curves in Figure 3, to facilitate the calculation [17]. The results, based on the macroscopic-model-simulated [^3^O_2_], [ROS]_rx, calc2_, are summarized in Figure 6b. Across the 20 patients (76 sites), the mean [ROS]_rx, calc2_ was 0.72 ± 0.20 mM (shown in Figure 6b). The [ROS]_rx, meas_ values based on the direct measurement of [^3^O_2_], served as our standard values, across 13 patients (21 sites), are summarized in Figure 6c. Across the entire 13-patient cohort spanning 21 distinct pleural cavity locations monitored, the mean [ROS]_rx, meas_ was 0.56 ± 0.26 mM. However, values ranged from a minimum of 0.31 mM to a maximum of 1.17 mM, representing a 3.8-fold variation in the reacted singlet oxygen levels generated during equal light dose delivery.

## 4. Discussion

This study represents the clinical implementation of ROS dosimetry during photodynamic therapy. By integrating multi-parametric monitoring of light fluence rate, photosensitizer concentration, and real-time tumor oxygenation fluctuations derived from diffuse correlation spectroscopy of tumor blood flow, comprehensive analysis of the [ROS]_rx_ was achieved in patients undergoing Photofrin-mediated pleural PDT. The key finding was the presence of substantial heterogeneities in [ROS]_rx_ levels across different regions within individual subjects as well as between patients receiving identical administered light doses. Although all patients received the same total prescribed light dose of 60 J/cm^2^, the computed [ROS]_rx_ values, using both measured and calculated [^3^O_2_], exhibited a similar pattern, revealing striking heterogeneities in the reactive oxygen species concentration among different patients.

Even when examining individual subjects, [ROS]_rx_ levels were highly non-uniform across the different measurement sites within the same patient’s pleural cavity during a given treatment session. The maximum intra-patient variability was 2.6-fold between the highest and lowest [ROS]_rx_ values at separate locations. These heterogeneities can be attributed to the combined influences of variable photosensitizer uptake across different tumor regions, as well as dynamically evolving changes in oxygenation status induced by PDT effects on a regional basis within the pleural cavity.

These observed variations clearly highlight a critical limitation of conventional dosimetry protocols that rely solely on light fluence and/or photosensitizer concentration as predictors of therapeutic PDT effects. The heterogeneities can be attributed to a combination of factors impacting the three key governing parameters of light fluence distribution, photosensitizer distribution, and spatiotemporal oxygenation dynamics within the pleural cavity tumor environment. Although a constant light fluence was delivered to the selected treatment sites in this study, differences in regional photosensitizer uptake, microenvironmental consumption, and vascular effects inevitably led to variable production of [ROS]rx. Moreover, the uneven distribution of light fluence across the entire inner pleural surface tends to exacerbate these variations in [ROS]_rx_ generation. Importantly, the dosimetry approach employed in this study enabled the quantitative identification of these heterogeneities, which would have been overlooked using standard techniques.

The ability to quantify [ROS]_rx_ distributions and identify under- or over-treated regions is valuable for reviewing treatment outcomes. Importantly, it also holds significant potential for optimizing PDT efficacy while minimizing off-target toxicities. In previous preclinical studies, [ROS]_rx_ has shown promise as a more accurate predictor of long-term tumor response compared to light fluence-based dosimetry [18]. Through further development of the dosimetry system, interventional guidance can be provided for adaptive light painting to homogenize the cytotoxic effects. This could dramatically improve therapeutic outcomes for a wide variety of solid tumor indications amenable to PDT. Computation of [ROS]_rx_ values utilized established ROSED modeling frameworks incorporating validated photochemical parameters. While some previous studies employed direct singlet oxygen luminescence detection techniques, the optical signal is extremely weak and fundamentally challenging to implement clinically on a multichannel level [16,31]. In contrast, the ROSED methodology represents a practical macroscopic approach retaining biological relevance. The study’s sizeable patient cohort, encompassing data from 13 subjects across 21 distinct measurement locations within the pleural cavity, reinforces the robustness and potential widespread applicability of the integrated dosimetry platform. The protocols are readily transferable, setting the stage for broader validation correlating [ROS]_rx_ with long-term treatment outcomes in future larger patient cohorts and potential expansion to other PDT indications beyond pleural tumors.

**Figure 7:**
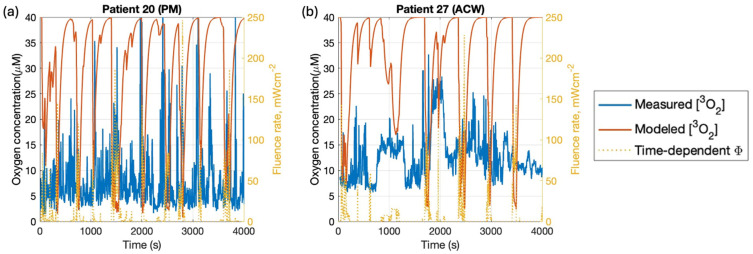
Comparison of simulated and measured oxygen concentrations over time for two representative cases, (**a**) #20 and (**b**) #27. The measured [^3^O_2_] is represented by the blue solid curve. The macroscopic model-simulated [^3^O_2_] using the light fluence data is represented by the red solid line. The time-dependent light fluence, ϕ, utilized by the model is represented by the yellow dotted line for reference.

**Figure 8:**
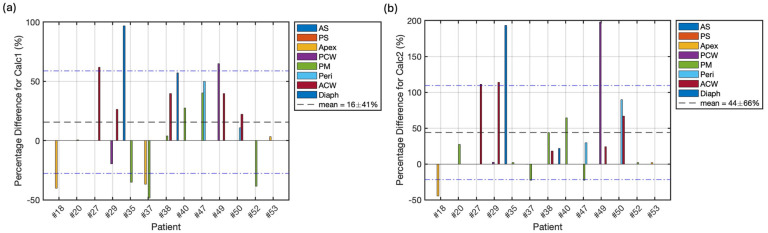
(**a**) Percentage difference between [ROS]_rx, calc1_ and [ROS]_rx, meas,_ where the average percentage difference is 16 ± 41%. (**b**) Percentage difference between [ROS]_rx, calc2_ and [ROS]_rx, meas,_ where the average percentage difference is 44 ± 66%. The mean and standard deviation are represented by the black dashed line and blue dash-dotted line respectively.

Under certain circumstances, like the limitation of the surgical condition, DCS may not be employed and eventually causes measurement of oxygen concentration failure. For the first time, we tried to overcome this limitation by developing two methods: (1) utilizing the averaged ${CF}_{O_{2}}$, summarized from available sites, and the PDT doses to calculate the [ROS]_rx, calc_, and (2) utilizing macroscopic modeling to for [^3^O_2_] simulation. The comparison of simulated and measured oxygen concentrations over time for a representative patient is shown in Figure 7. The measured and simulated outcomes exhibit distinct patterns, where the measured [^3^O_2_] exhibits peaks corresponding to the approaching point light source, while the simulated [^3^O_2_] tends to decrease when light is applied, which agrees with our ROSED model (Equations (5)–(7)), suggesting the consumption of ground-state oxygen during PDT. The difference can be attributed to the assumption of constant blood flow in the model, whereas variations in blood flow can significantly influence the tissue oxygen level, in addition to illumination [32,33]. The assumption of a high initial oxygen concentration (40 mM) leads to significantly higher overall results when calculated using method 2. The statistical differences of [ROS]_rx, calc1_ and [ROS]_rx, calc2_ compared to [ROS]_rx, meas_ are summarized in Figure 8a,b, respectively. The mean percentage differences of 16 ± 41% for the average ${CF}_{O_{2}}$ method and 44 ± 66% for the macroscopic modeling method indicate that method 1 (average ${CF}_{O_{2}}$ method) demonstrates superior accuracy and precision. This suggests that method 1 may serve as a valuable tool for providing important insights by filling in missing data with greater simplicity. While the macroscopic method was less accurate in this study, it could potentially be improved through further investigation of a more appropriate initial oxygen constant for clinical use, potentially offering insights into internal biomedical processes during treatment. For future clinical applications where direct oxygen measurement is not feasible, the average method may serve as a viable alternative, particularly in settings where simplicity and reliability are paramount. These findings underscore the importance of method selection in oxygen concentration analysis and suggest avenues for future research to optimize both approaches for clinical use.

Our current ROSED implementation is inclusive of both type I and II PDT mechanisms utilizing the fundamental approach of combining light dosimetry, photosensitizer concentration monitoring, and tissue oxygenation measurements. It can be adapted for not only type II photosensitizers (such as Photofrin) but also ROS species involved in Type I PDT mechanisms. Type I processes involve electron transfer reactions generating superoxide anion radicals, hydroxyl radicals, and hydrogen peroxide [10,34,35]. While different detection methods would be needed for these ROS species, the general framework of explicit dosimetry incorporating real-time physiological measurements can be valuable for optimizing treatment outcomes across various PDT approaches. Future studies can explore adapting our platform for photosensitizers like chlorins or phthalocyanines that can generate both Type I and Type II photochemical reactions [36,37,38].

**Figure 9:**
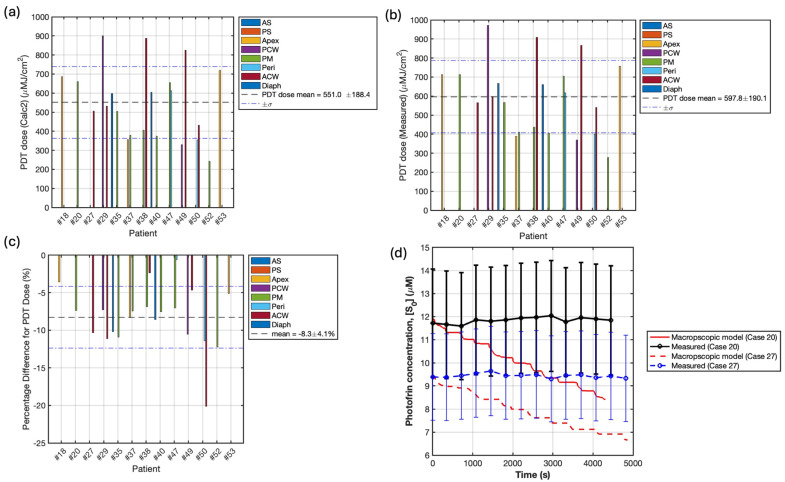
PDT doses for 13 cases using Equation (1) calculated using (**a**) our macroscopic model and (**b**) measured data, where the mean PDT doses are 551.0 ± 188.4 μMJ/cm^2^ and 597.8 ± 190.1, respectively. (**c**) Percentage difference between PDT dose (calc 2) and PDT dose (measured), where the average percentage difference is −8.3 ± 4.1%. (**d**) Comparison of the measured and model-simulated photosensitizer concentrations during treatments for two representative cases. The black solid curve represents the measured data for case 20. The red solid curve represents the simulated result for case 20. The blue and red dash curves represent the measured and simulated data for case 27. The simulated results demonstrate a declined trend during treatment, while the measure data remains constant.

Figure 8 shows the percentage difference of [ROS]_rx,calc1_ and [ROS]_rx,calc2_ compared to the measured [ROS]rx,meas for all 13 patients. It shows that [ROS]_rx,calc1_ agree with measurement to within 16%, much smaller than that based on finite element modeling (calc2), with 44%. As shown in Figure 7, calc2 overestimates the tissue oxygenation. However, our finite element model (calc2) can correctly calculate PDT dose as shown in Figure 9c.

Figure 9 provides further insights into the comparison between measured and calculated PDT doses, as well as the dynamics of photosensitizer concentration during treatment. Figure 9a,b illustrate the PDT doses calculated using our macroscopic model and measured data, respectively, for 13 cases. The mean PDT doses were found to be 551.0 ± 188.4 μM·J/cm² for the model-based calculations and 597.8 ± 190.1 μM·J/cm² for the measured data. This close agreement between modeled and measured PDT doses, with only an 8.3 ± 4.1% average difference (Figure 9c), underscores the potential accuracy of our macroscopic model in predicting PDT dose delivery. However, Figure 9d reveals an interesting discrepancy between measured and simulated photosensitizer concentrations during treatments. While the measured data shows relatively constant photosensitizer levels throughout the treatment, our model predicts a gradual decline, which may explain the lower PDT dose values obtained using the macroscopic model. This difference highlights a key area for future investigation and model refinement. The constant measured photosensitizer concentration suggests that photobleaching may be less significant in clinical settings than previously assumed, or that there might be ongoing replenishment of the photosensitizer during treatment. Understanding this discrepancy could lead to improved dosimetry models and potentially more accurate predictions of treatment outcomes.

However, it is crucial to note that there is typically a large uncertainty associated with in vivo photosensitizer concentration measurements. This uncertainty arises from various factors, including tissue heterogeneity, variations in optical properties, and limitations of the measurement techniques themselves [39,40,41,42]. Given these substantial uncertainties, the difference between the measured and modeled photosensitizer concentrations may not necessarily indicate a flaw in the model [43,44]. The apparent stability in measured photosensitizer levels could be partially attributed to measurement uncertainties masking subtle changes, while the model’s prediction of a gradual decline aligns with the expected photobleaching effect. This highlights the complexity of translating theoretical models to clinical settings and underscores the need for further refinement of both measurement techniques and modeling approaches. Future work should focus on reducing measurement uncertainties and incorporating these uncertainties into the model to provide a more comprehensive understanding of photosensitizer dynamics during PDT treatments.

There are, however, some limitations to the current work that warrant further investigation. First, the conversion of the diffuse correlation spectroscopy blood flow index to approximate oxygen concentration utilized a single scaling factor based on previous preclinical studies. While consistent with reported values, this conversion could potentially vary between different tumor types and populations. Direct comparison to gold standard techniques, like oxygen electrodes, is needed to validate and potentially develop more advanced predictive models incorporating additional parameters beyond just blood flow. Additionally, the ROSED calculations were based on established macroscopic modeling frameworks making certain assumptions about parameter constancy and incorporating empirical corrections. Looking ahead, explicit microscopic simulations directly modeling particle transport using powerful computational modeling tools, like COMSOL Multiphysics^®^, could provide higher resolution [ROS]_rx_ mapping, albeit at significantly greater computational expense. An interesting avenue would be to combine the efficiency of ROSED with high-fidelity computational modeling results to develop more accurate hybrid dosimetry models. Another potential limitation is the specificity of the current study to a particular PDT regimen–Photofrin-mediated treatment of pleural mesothelioma. While the general principles of multi-parametric dosimetry should apply broadly, the specific dosimetry implementation, instrumentation, and calibration procedures would require tailoring for other photosensitizer drugs and tumor indications based on their unique photochemical characteristics. Further studies are needed to establish [ROS]_rx_ quantification protocols across the diverse range of clinical PDT applications.

In summary, this work successfully implemented the measurement and analysis of explicit [ROS]_rx_ dosimetry during pleural PDT, revealing substantial heterogeneities that could impact long-term therapeutic outcomes. The integrated multi-modal dosimetry platform combining light tracking, photosensitizer monitoring, and non-invasive tumor oxygenation measurements represents a pioneering technological advance. It is important to note that our ROSED measurements quantify both singlet oxygen generation through Type II photochemical pathways and type I photochemical pathways. The Type I pathway-generating superoxide anions, hydrogen peroxide, and hydroxyl radicals may contribute to the overall therapeutic effect, particularly in regions of lower oxygen tension where Type II reactions are limited. Furthering the development of this system by incorporating a real-time light fluence navigation system would facilitate the realization of a comprehensive treatment planning system, enabling an unprecedented level of personalization and optimization in PDT dose deposition. Moreover, the expansion of the [ROS]_rx_ system to encompass a full eight channels is a desirable prospect, as it would allow for the coverage of a broader range of locations, thereby aiding in the pursuit of the ultimate objective: achieving a uniform [ROS]_rx_ distribution for pleural PDT.

## 5. Conclusions

The present work represents a comprehensive clinical investigation into ROSED implemented clinically and constitutes the first exploration into the feasibility of real-time monitoring for patients undergoing Photofrin-mediated pleural PDT. The key finding was that [ROS]_rx_ exhibited substantial heterogeneities across the measurement sites, with up to 3.8-fold variations between patients and 2.6-fold differences within each individual’s pleural cavity. This is despite all patients receiving the same prescribed light dose of 60 J/cm^2^, highlighting a critical limitation of conventional light fluence-based dosimetry protocols. The observed heterogeneities in [ROS]_rx_ can be attributed to the combined impacts of variable photosensitizer uptake and dynamically fluctuating physiological parameters, such as tissue oxygenation and light fluence distribution, during the PDT treatment. A further crucial finding is the utilization of the calculated ground-state oxygen concentration in the [ROS]_rx_ calculation, which enables broader applicability of our system when direct oxygenation measurement is precluded during photodynamic therapy (PDT) due to complexities arising from clinical conditions. These findings underscore the crucial need and possibility for explicit dosimetry techniques that directly monitor the effective PDT quantity, i.e., [ROS]_rx_.

Moving forward, our primary objective is the seamless integration of light dosimetry, photosensitizer fluorescence monitoring, and non-invasive diffuse correlation spectroscopy measurements of tumor blood flow into a unified treatment monitoring and planning platform. The ROSED system will be expanded to incorporate eight measurement channels, facilitating comprehensive mapping of [ROS]_rx_ distribution throughout the pleural cavity. Crucially, this enhanced system will be coupled with an intraoperative navigation system, enabling the precise spatial tracking and mapping of the [ROS]_rx_ measurements within the anatomical context of each patient’s pleural cavity. This integrated approach aims to develop a real-time adaptive treatment planning strategy to achieve uniform [ROS]_rx_ distributions and optimize therapeutic outcomes for pleural PDT.

## Figures and Tables

**Table 1:** Photochemical parameters obtained from the literature.

Parameter	Definition	Value
σ	Specific photobleaching ratio	$7.6\times 10^{-5}\mu M^{-1}$
g	Macroscopic maximum oxygen supply rate	0.76 $\mu Ms^{-1}$
δ	Low concentration correction	33 μM
ξ	Specific oxygen consumption rate	$3.7\times 10^{-3}{cm}^{2}s^{-1}mW^{-1}$
β	Oxygen quenching threshold concentration	11.9 μM

**Table 2:** Summary of monitored sites for 20 patients when good data are obtained for ROSED. The table shows the number of channels used for light dosimetry, PDT dose dosimetry, and DCS blood flow monitoring for each patient.

Patient *	Light Dosimetry (Channels)	PDT Dose Dosimetry (Channels)	DCS Blood Flow Monitoring (Channels)
07	8	2	0
08	8	2	0
12	8	2	0
14	8	2	0
16	8	2	0
17	8	4	0
18	8	4	1
20	8	4	1
27	8	4	1
29	8	4	2
32	8	4	0
35	8	4	2
37	8	4	2
38	8	4	2
40	8	4	2
47	8	4	2
49	8	4	2
50	8	4	2
52	8	8	1
53	8	8	1

* In our randomized study, patients 09, 10, 11, 13, 15, 19, 21, 22, 24, 25, 26, 28, 30, 31, 33, 36, 39, 41, 42, 43, 44, 45, 46, and 48 had surgery alone without PDT. Other Patients were not included due to various reasons: not treated at UPenn (23), PDT not completed (34), or never started in the clinical protocol (51).

## Data Availability

The raw data supporting the conclusions of this article will be made available by the authors on request.

## Supplementary Materials

The following supporting information can be downloaded at: https://www.mdpi.com/article/10.3390/antiox13121436/s1, Table S1: CONSORT 2010 checklist of information to include when reporting a randomised trial.

## References

[1] Prazmo E.J., Kwasny M., Lapinski M., Mielczarek A. (2016). Photodynamic Therapy As a Promising Method Used in the Treatment of Oral Diseases. Adv. Clin. Exp. Med..

[2] Kwiatkowski S., Knap B., Przystupski D., Saczko J., Kedzierska E., Knap-Czop K., Kotlinska J., Michel O., Kotowski K., Kulbacka J. (2018). Photodynamic therapy-mechanisms, photosensitizers and combinations. Biomed. Pharmacother..

[3] Dudzik T., Domanski I., Makuch S. (2024). The impact of photodynamic therapy on immune system in cancer—An update. Front. Immunol..

[4] Moghissi K., Dixon K. (2008). Update on the current indications, practice and results of photodynamic therapy (PDT) in early central lung cancer (ECLC). Photodiagnosis Photodyn. Ther..

[5] Lin L., Song X., Dong X., Li B. (2021). Nano-photosensitizers for enhanced photodynamic therapy. Photodiagnosis Photodyn. Ther..

[6] Barr H., Bown S.G., Krasner N., Boulos P.B. (1989). Photodynamic therapy for colorectal disease. Int. J. Color. Dis..

[7] Pignatelli P., Umme S., D’Antonio D.L., Piattelli A., Curia M.C. (2023). Reactive Oxygen Species Produced by 5-Aminolevulinic Acid Photodynamic Therapy in the Treatment of Cancer. Int. J. Mol. Sci..

[8] Ding Y., Pan Q., Gao W., Pu Y., Luo K., He B. (2023). Reactive oxygen species-upregulating nanomedicines towards enhanced cancer therapy. Biomater. Sci..

[9] Nonell S., Flors C. (2016). Singlet Oxygen: Applications in Biosciences and Nanosciences.

[10] Castano A.P., Demidova T.N., Hamblin M.R. (2004). Mechanisms in photodynamic therapy: Part one-photosensitizers, photochemistry and cellular localization. Photodiagnosis Photodyn. Ther..

[11] Roberts J.E. (2020). Techniques to Improve Photodynamic Therapy. Photochem. Photobiol..

[12] Yassine A.A., Lilge L., Betz V. (2019). Optimizing interstitial photodynamic therapy with custom cylindrical diffusers. J. Biophotonics.

[13] Herr H., Cho H.J., Yu S. (2007). Burns caused by accidental overdose of photochemotherapy (PUVA). Burns.

[14] Sun H., Ong Y., Yang W., Sourvanos D., Dimofte A., Busch T.M., Singhal S., Cengel K.A., Zhu T.C. (2024). Clinical PDT dose dosimetry for pleural Photofrin-mediated photodynamic therapy. J. Biomed. Opt..

[15] Pogue B.W., Elliott J.T., Kanick S.C., Davis S.C., Samkoe K.S., Maytin E.V., Pereira S.P., Hasan T. (2016). Revisiting photodynamic therapy dosimetry: Reductionist & surrogate approaches to facilitate clinical success. Phys. Med. Biol..

[16] Jarvi M.T., Niedre M.J., Patterson M.S., Wilson B.C. (2006). Singlet oxygen luminescence dosimetry (SOLD) for photodynamic therapy: Current status, challenges and future prospects. Photochem. Photobiol..

[17] Wang K.K., Finlay J.C., Busch T.M., Hahn S.M., Zhu T.C. (2010). Explicit dosimetry for photodynamic therapy: Macroscopic singlet oxygen modeling. J. Biophotonics.

[18] Penjweini R., Kim M.M., Ong Y.H., Zhu T.C. (2020). ^1^O_2_ determined from the measured PDT dose and ^3^O_2_ predicts long-term response to Photofrin-mediated PDT. Phys. Med. Biol..

[19] Betrouni N., Munck C., Bensoltana W., Baert G., Dewalle-Vignion A.S., Scherpereel A., Mordon S. (2017). Real-time light dosimetry for intra-cavity photodynamic therapy: Application for pleural mesothelioma treatment. Photodiagnosis Photodyn. Ther..

[20] van Doeveren T.E.M., van Veen R.L.P., van den Boom F., Tan I.B., Schreuder W.H., Karakullukcu M.B. (2020). Intra-cavity Photodynamic Therapy for malignant tumors of the paranasal sinuses: An in vivo light dosimetry study. Photodiagnosis Photodyn. Ther..

[21] Middelburg T.A., Hoy C.L., Neumann H.A., Amelink A., Robinson D.J. (2015). Correction for tissue optical properties enables quantitative skin fluorescence measurements using multi-diameter single fiber reflectance spectroscopy. J. Dermatol. Sci..

[22] Baran T.M., Wilson J.D., Mitra S., Yao J.L., Messing E.M., Waldman D.L., Foster T.H. (2012). Optical property measurements establish the feasibility of photodynamic therapy as a minimally invasive intervention for tumors of the kidney. J. Biomed. Opt..

[23] Wen B., Urano M., O’Donoghue J.A., Ling C.C. (2006). Measurements of partial oxygen pressure pO2 using the OxyLite system in R3327-AT tumors under isoflurane anesthesia. Radiat. Res..

[24] Shang Y., Li T., Yu G. (2017). Clinical applications of near-infrared diffuse correlation spectroscopy and tomography for tissue blood flow monitoring and imaging. Physiol. Meas..

[25] Busch D.R., Balu R., Baker W.B., Guo W., He L., Diop M., Milej D., Kavuri V., Amendolia O., St Lawrence K. (2019). Detection of Brain Hypoxia Based on Noninvasive Optical Monitoring of Cerebral Blood Flow with Diffuse Correlation Spectroscopy. Neurocrit. Care.

[26] Yu G., Durduran T., Zhou C., Wang H.W., Putt M.E., Saunders H.M., Sehgal C.M., Glatstein E., Yodh A.G., Busch T.M. (2005). Noninvasive monitoring of murine tumor blood flow during and after photodynamic therapy provides early assessment of therapeutic efficacy. Clin. Cancer Res..

[27] Ong Y.H., Dimofte A., Kim M.M., Finlay J.C., Sheng T., Singhal S., Cengel K.A., Yodh A.G., Busch T.M., Zhu T.C. (2020). Reactive Oxygen Species Explicit Dosimetry for Photofrin-mediated Pleural Photodynamic Therapy. Photochem. Photobiol..

[28] Kim M.M., Zhu T.C., Ong Y.H., Finlay J.C., Dimofte A., Singhal S., Glatstein E., Cengel K.A. (2020). Infrared navigation system for light dosimetry during pleural photodynamic therapy. Phys. Med. Biol..

[29] Saczko J., Kulbacka J., Chwilkowsa A., Pola A., Lugowski M., Marcinkowska A., Malarska A., Banas T. (2007). Cytosolic superoxide dismutase activity after photodynamic therapy, intracellular distribution of Photofrin II and hypericin, and P-glycoprotein localization in human colon adenocarcinoma. Folia Histochem. Cytobiol..

[30] Orenstein A., Kostenich G., Roitman L., Shechtman Y., Kopolovic Y., Ehrenberg B., Malik Z. (1996). A comparative study of tissue distribution and photodynamic therapy selectivity of chlorin e6, Photofrin II and ALA-induced protoporphyrin IX in a colon carcinoma model. Br. J. Cancer.

[31] Bresoli-Obach R., Torra J., Zanocco R.P., Zanocco A.L., Nonell S. (2021). Singlet Oxygen Quantum Yield Determination Using Chemical Acceptors. Methods Mol. Biol..

[32] Causin P., Guidoboni G., Malgaroli F., Sacco R., Harris A. (2016). Blood flow mechanics and oxygen transport and delivery in the retinal microcirculation: Multiscale mathematical modeling and numerical simulation. Biomech. Model. Mechanobiol..

[33] Albright A., Fry B.C., Verticchio A., Siesky B., Harris A., Arciero J. (2023). Metabolic blood flow regulation in a hybrid model of the human retinal microcirculation. Math. Biosci..

[34] Mroz P., Pawlak A., Satti M., Lee H., Wharton T., Gali H., Sarna T., Hamblin M.R. (2007). Functionalized fullerenes mediate photodynamic killing of cancer cells: Type I versus Type II photochemical mechanism. Free Radic. Biol. Med..

[35] Ayaz F., Yetkin D., Yuzer A., Demircioglu K., Ince M. (2022). Non-canonical anti-cancer, anti-metastatic, anti-angiogenic and immunomodulatory PDT potentials of water soluble phthalocyanine derivatives with imidazole groups and their intracellular mechanism of action. Photodiagnosis Photodyn. Ther..

[36] Plaetzer K., Krammer B., Berlanda J., Berr F., Kiesslich T. (2009). Photophysics and photochemistry of photodynamic therapy: Fundamental aspects. Lasers Med. Sci..

[37] Yao L., Xie S., Liu Y., Mengqi L., Xia J., Lu B. (2024). Singlet oxygen storage and controlled release for improving photodynamic therapy against hypoxic tumor. Chem. Commun..

[38] Pal K., Singh S., Itakura S., Hashimoto M., Kusamori K., Nishikawa M. (2024). Reactive oxygen species augmented polydopamine-chlorin e6 nanosystem for enhanced chemo/photothermal/photodynamic therapy: A synergistic trimodal combination approach in vitro & in vivo. Int. J. Biol. Macromol..

[39] Silvestri R., Rea F., Vitiello M., Moretti G., Aprile V., Lucchi M., Aretini P., Mazzanti C.M., Landi S., Gemignani F. (2024). Comparative analysis of genetic variants in pleural fluids and solid tissue biopsies of pleural mesothelioma patients: Implications for molecular heterogeneity assessment. Heliyon.

[40] Nandy S., Mostafa A., Kumavor P.D., Sanders M., Brewer M., Zhu Q. (2016). Characterizing optical properties and spatial heterogeneity of human ovarian tissue using spatial frequency domain imaging. J. Biomed. Opt..

[41] Goyal A., van den Wijngaard J., van Horssen P., Grau V., Spaan J., Smith N. Intramural spatial variation of optical tissue properties measured with fluorescence microsphere images of porcine cardiac tissue. Proceedings of the 2009 Annual International Conference of the IEEE Engineering in Medicine and Biology Society.

[42] Lee C.C., Pogue B.W., O’Hara J.A., Wilmot C.M., Strawbridge R.R., Burke G.C., Hoopes P.J. (2003). Spatial heterogeneity and temporal kinetics of photosensitizer (AlPcS2) concentration in murine tumors RIF-1 and MTG-B. Photochem. Photobiol. Sci..

[43] Galstyan A., Strokov K. (2022). Influence of photosensitizer concentration and polymer composition on photoinduced antimicrobial activity of PVA- and PVA-chitosan-based electrospun nanomaterials cross-linked with tailor-made silicon(IV) phthalocyanine. Photochem. Photobiol. Sci..

[44] Balhaddad A.A., AlQranei M.S., Ibrahim M.S., Weir M.D., Martinho F.C., Xu H.H.K., Melo M.A.S. (2020). Light Energy Dose and Photosensitizer Concentration Are Determinants of Effective Photo-Killing against Caries-Related Biofilms. Int. J. Mol. Sci..

